# Carcinome épidermoïde de la vulve chez une patiente infectée par le VIH-1 en échec de traitement antirétroviral de première ligne

**DOI:** 10.11604/pamj.2020.36.181.20628

**Published:** 2020-07-14

**Authors:** Alassane Dièye, Bruce Wembulua Shinga, Jacques Noel Tendeng, Moustapha Diedhiou, Birame Seck, Abdourahmane Ndong, Ndèye Méry Dia-Badiane

**Affiliations:** 1 UFR des Sciences de la Santé, Université Gaston Berger de Saint-Louis, Saint-Louis, Sénégal,; 2 Service des Maladies Infectieuses et Tropicales du CHNU de Fann, Université Cheikh Anta Diop, Dakar, Sénégal,; 3 Service de Chirurgie Générale, Centre Hospitalier Régional de Saint-Louis, Saint-Louis, Sénégal

**Keywords:** Carcinome épidermoïde, vulve, VIH, Squamous cell carcinoma, vulva, HIV

## Abstract

Le cancer de la vulve est une affection rarement rapportée dans la littérature. Chez la femme jeune, il est le plus souvent lié à l´infection par le papillomavirus humain (HPV) alors que chez les femmes ménopausées, chez qui ce cancer est plus fréquent, il serait lié à la carence œstrogénique. En outre, l´infection à VIH augmente le risque de survenue chez les femmes séropositives de néoplasies vulvaires du fait de la prévalence élevée de l´infection à HPV chez elles. Ainsi devant toute lésion suspecte de la vulve, une biopsie suivie d´un examen anatomo-pathologique devra être réalisée afin de poser le diagnostic. Nous rapportons le cas d´un carcinome épidermoïde de la vulve chez une patiente séropositive au VIH-1 en échec de traitement antirétroviral (ARV) de première ligne.

## Abstract (en)

Vulvar cancer has been rarely reported in the literature. In young women, it is most often caused by human papillomavirus (HPV), whereas in postmenopausal women, in whom this cancer is more common, it would be caused by estrogen deficiency. Moreover, HIV infection increases the risk of developing vulvar cancer in HIV-positive women as a consequence of the high prevalence of HPV infection in these subjects. Thus, in patients with suspected vulva lesion, biopsy followed by anatomo-pathological examination should be performed in order to establish the diagnosis. We here report a case of vulvar squamous cell carcinoma in a HIV-1-positive patient with first-line antiretroviral therapy (ARV) failure.

## Introduction

Le cancer de la vulve est une entité rare, secondaire le plus souvent lié à une infection par le human papilloma virus (HPV) chez la femme jeune [1], alors que chez la femme ménopausée la carence œstrogénique expliquerait sa genèse [2]. Il est le 4^e^type de cancer gynécologique le plus répandu après les cancers du col, de l´endomètre et des ovaires et englobe environ 6% de toutes les tumeurs malignes des voies génitales féminines [3]. Le carcinome épidermoïde de la vulve constitue la variété histologique la plus fréquente [4]. L´infection à VIH augmente le risque de survenue chez les femmes séropositives de cancers de la vulve [5] du fait de la forte prévalence de l´infection à HPV chez elles [6]. Nous rapportons ainsi le cas d´un carcinome épidermoïde de la vulve chez une patiente infectée par le VIH-1 en échec de traitement ARV de première ligne dont l´évolution a été marquée par le décès dans un tableau de choc septique.

## Patient et observation

**Figure 1:**
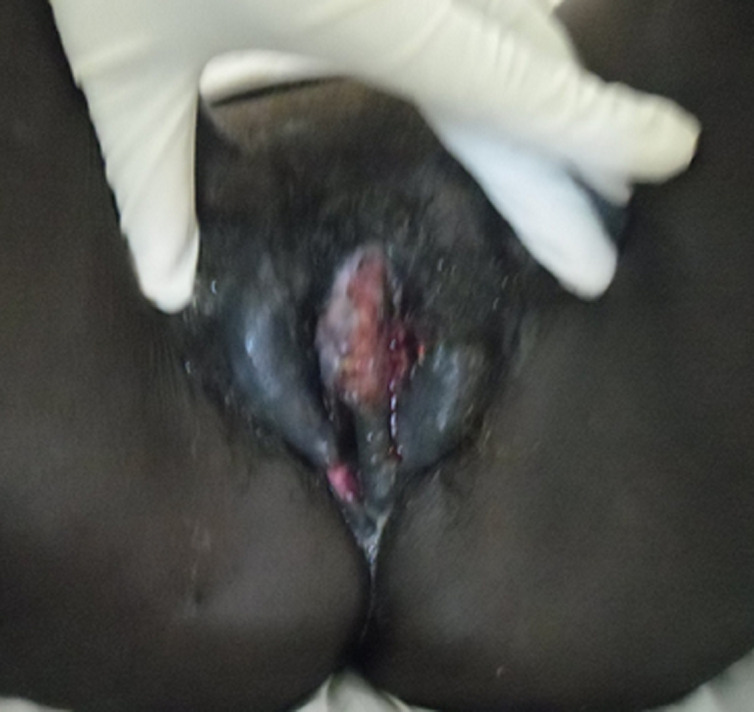
carcinome épidermoïde ulcéro-bourgeonnante de la vulve

Il s´agit d´une patiente de 45 ans séropositive au VIH-1, suivie depuis mai 2013 avec un taux de lymphocytes TCD4+ initial=14cells/mm^3^sous TDF-3TC-EFV. Elle avait fait un échec thérapeutique suite à une mauvaise observance thérapeutique avec à l´hospitalisation un taux de lymphocytes TCD4+ effondré à 01 cell/mm^3^. Elle avait consulté pour une toux quinteuse productive ramenant des expectorations blanchâtres évoluant depuis deux semaines, une dyspnée d´installation progressive, une fièvre sans horaire fixe et une altération sévère de l´état général. L´examen physique retrouvait: une condensation pulmonaire bilatérale compliquée de détresse respiratoire avec une FR=52 cycles/min avec une désaturation (SaO2=75% à l´air ambiant), un syndrome d´immunodépression clinique, un syndrome anémique et une lésion ulcéro-bourgeonnante, étendue sur les grandes et petites lèvres, dure, irrégulière, saignant facilement au contact et malodorante (Figure 1) avec une adénopathie inguinale satellite non inflammatoire. On notait une température de 38,2°C avec une tachycardie=132 battements/mn en rapport et un collapsus cardio-vasculaire (TA=88/59mmHg). La biologie montrait: une leucopénie=2000/uL; hémoglobine=8,5g/L; glycémie à jeûn=0,92g/L. Le bilan rénal, hépatique ainsi que l´ionogramme sanguin était normal. La radiographie du thorax, le bilan tuberculeux, l´antigénémie cryptococcique, la sérologie toxoplasmique n´étaient pas réalisés faute de moyens. L´examen anatomo-pathologique après biopsie de la lésion vulvaire objectivait un carcinome épidermoïde infiltrant kératinisé de la vulve. Le bilan d´extension de la tumeur n´était pas réalisé faute de moyens. Malgré l´antibiothérapie et les mesures de réanimation, la patiente décède au 2^e^jour d´hospitalisation dans un tableau de choc septique.

## Discussion

Le cancer de la vulve constitue le 4^e^type de cancer gynécologique le plus répandu après les cancers du col, de l´endomètre et des ovaires. C´est une entité rare qui englobe environ 6% de toutes les tumeurs malignes des voies génitales féminines. On note une augmentation de son incidence avec l´âge [7]. Le carcinome vulvaire touche principalement les femmes âgées [3]. Selon les statistiques sur le cancer, il y aurait plus de 6.000 cas et 1.150 décès ont été enregistrés chaque année aux États-Unis [8]. Bien que l´incidence du cancer de la vulve soit faible, elle a augmenté au cours des dernières décennies, en particulier chez les jeunes femmes. Chez ces dernières, il est rapporté que l´infection génitale persistante par le HPV était la cause essentielle du développement de néoplasies vulvaires [1]. Chez les femmes séropositives, l´immunodéficience induite par le VIH favorise la survenue et la persistance de l´infection par le HPV conduisant ainsi plus facilement au développement de cancers génitaux notamment de la vulve [9]. En effet, les femmes infectées par le VIH contrôlent beaucoup moins bien l´infection par le HPV que les femmes non infectées [10] et, parmi les femmes infectées par le VIH, celles ayant un nombre de lymphocytes T-CD4+ inférieur à 200 cells/mm^3^ont moins de chance (réduction de 71% par rapport aux femmes ayant un nombre de lymphocytes TCD4+ supérieur à 500cells/mm^3^) d´éliminer une infection par le HPV que les femmes ayant un nombre de lymphocytes T-CD4+ compris entre 200 et 500 cells/mm^3^(réduction de 32% par rapport aux femmes ayant un nombre de lymphocytes TCD4+ supérieur à 500 cells/mm^3^) [10].

Selon le type histologique, il s´agit essentiellement (90%) de cancer épidermoïde et rarement de mélanome, d´adénocarcinome des glandes de Bartholin ou de tumeurs cutanées. Les cancers épidermoïdes sont, comme les cancers du col et du vagin, précédés par des lésions de type néoplasie intra-épithéliales (VIN, *vulvar intraepithelial neoplasia* ou néoplasie intraépithéliale vulvaire), dont la transformation en cancer invasif est de l´ordre de 5 à 10%, qu´il s´agisse de papulose bowénoïde, de verrues génitales ou de maladie de Bowen [4]. Notre patiente avait un carcinome épidermoïde kératinisant de la vulve. La tumeur est le plus souvent localisée sur les grandes lèvres (80%), suivies des petites lèvres (14,3%), et le clitoris (5,7%) [3]. Notre patiente avait une néoplasie étendue de toute la vulve (Figure 1). Cliniquement, le carcinome épidermoïde de la vulve peut se manifester par une irritation chronique vulvaire, un prurit vulvaire, une sensation de brûlure au niveau des lèvres génitales, une dyspareunie ou encore la présence de zones décolorées sur les lèvres. Il est donc nécessaire de procéder à un examen gynécologique munitieux chez les femmes séropositives afin de dépister précocement des cancers génitaux [11]. Des métastases restent possibles et se font via le système lymphatique vers la région inguinale ou fémorale mais aussi à travers les ganglions lymphatiques pelviens et distants. Cependant, la propagation hématogène est inhabituelle [12]. Les facteurs de mauvais pronostic sont: l´extension ganglionnaire lymphatique, la taille de la tumeur et les récidives tumorales [13].

Le pronostic du cancer de la vulve semble meilleur chez les femmes présentant une infection chronique par le HPV [14]. La chirurgie radicale garde toute sa place même en cette ère de conservation d´organes. La vulvectomie radicale associée au curage ganglionnaire inguinal et à la radiothérapie post-opératoire [15]. Cependant, le taux de mortalité péri-opératoire associé avec cette chirurgie peut aller jusqu´à 10% et le taux d´incidence de complications est supérieur à 66,6%. De plus, il comporte un risque de morbidité liée à la procédure, de défiguration physique, sexuelle, de dysfonctionnement, et une influence largement inconnue sur l´ensemble qualité de vie. Son taux de survie à 5 ans est inférieur à 50% [16]. La radiothérapie post-opératoire est pratiquée devant des facteurs de risque de récidive locale: un volume tumoral supérieur à 4cm; une marge chirurgicale étroite (moins de 8mm) et un envahissement vasculaire lymphatique profond et le statut ganglionnaire positif [8]. Pour l´apport de la chimiothérapie surtout à base de cisplatine ou de mitomycine, de 5FU; des régressions tumorales complètes ont été observées dans 30 à 50% des cas dans des séries rétrospectives. Cependant, aucun essai randomisé n´a pu être réalisé à ce jour pour évaluer l´efficacité de la RCC (radiothérapie et chimiothérapie concomitante) comparée à la radiothérapie seule en raison du petit nombre de patientes. La curiethérapie est d´indication rare, c´est surtout dans le traitement des reliquats tumoraux au contact de l´urètre ou dans le tiers inférieur du vagin ou lors des traitements conservateurs des tumeurs de moins de 3cm [7]. La chimio-radiothérapie constitue une autre alternative thérapeutique mais n´est pas cependant dénuée d´effets secondaires (toxicité cutanée sévère de la vulve et du périnée) [17].

## Conclusion

Le cancer de la vulve est le 4^e^type de cancer gynécologique le plus fréquent. La forte prévalence de l´infection par le HPV au cours du VIH/Sida expose à un risque accru de cancers génitaux notamment vulvaires. Une biopsie avec examen anamo-pathologique doit être réalisée devant toute lésion suspecte de la vulve chez les patientes séropositives afin de poser un diagnostic précoce.
